# Interrelationship between tetracycline resistance determinants, phylogenetic group affiliation and carriage of class 1 integrons in commensal *Escherichia coli* isolates from cattle farms

**DOI:** 10.1186/s12917-018-1661-3

**Published:** 2018-11-12

**Authors:** Kuastros Mekonnen Belaynehe, Seung Won Shin, Han Sang Yoo

**Affiliations:** Department of Infectious Diseases, College of Veterinary Medicine, Seoul National University, Seoul, 08826 Republic of Korea

**Keywords:** *E. coli*, Class 1 integrons, Tetracycline resistance, Phylogenetic group, PFGE

## Abstract

**Background:**

Carriage of antibiotic-resistant foodborne pathogens by food production animals is one of many contributors to treatment failure in health care settings, and it necessitates an integrated approach to investigate the carriage of resistant pathogens harboring integrons in food-producing animals.

**Methods:**

*Escherichia coli* isolates with reduced susceptibility to tetracycline antibiotics (*n* = 92) were tested for associations between carriage of class1 integrons, phylogenetic group affiliation and tetracycline resistance determinants using the MIC method, PFGE analysis, PCR and sequencing.

**Results:**

Phylogroups B1 and A were the most common (58.7 and 19.6%, respectively), followed by groups D (20.7%) and B2 (1.1%). All isolates carried at least one of the *tet* genes examined. In addition, 88 (95.7%) of all tetracycline-resistant isolates carried *tet(A)* or *tet(B)*, while 47 (51.1%) and 41 (44.6%) harbored only *tet(A)* or *tet(B)*, respectively. Likewise, isolates harboring these genes had a higher chance (*P* < 0.05) of carrying class 1 integrons. Of the tested isolates, 38 (41.3%) carried the *intI1* gene. Classical integrons with complete genes (*sul1* and *qacE∆1*) at the 3′-CS were recognized in 27 isolates. PCR screening and subsequent sequencing demonstrated that 84.2% (32/38) of the *intI1*-positive isolates harbored resistance gene cassettes. Overall, seven gene cassettes were identified, either solely or combined with another gene cassette. The most common gene was *aadA1* (10 isolates), followed by a combination of *aadA1-dfrA1* (seven isolates), *aadA1-dfrA12* (six isolates) and *aadA1-aadA2-dfrA12* (three isolates). Genetic typing using PFGE showed minimum clonal relatedness with 28 different clusters and 12–25 discernible DNA fragments.

**Conclusions:**

This study brings new insight into the relationships between the presence of integrons, phylogenetic group association and characteristics of tetracycline antibiotic resistance determinants in commensal *E. coli* strains.

## Background

The spread and emergence of resistance to antimicrobial drugs among bacteria has been observed over the past several decades, and this constraint has been a constant impediment to effective infectious disease therapy for as long as antibiotics have been used [1]. In many cases, multidrug resistance was determined to be associated with transmissible plasmids, and the importance of integrons in the acquisition of resistance genes constitute the major vector of multidrug resistance in Gram-negative and, to a lesser extent, in Gram-positive bacteria [2–4].

Over the last few years, rigorous exploration of the diversity of integrons in natural environments has indicated that they are more than just a curious feature of antibiotic-resistant pathogens but that they play a more general and crucial role in the genomic evolution and adaptation of bacteria [5]. To date, five mobile integron classes have been described and characterized based on variations in the *intI* sequences. However, class 1 integrons are ubiquitous and the most frequently encountered among clinical and commensal isolates; therefore, they have been the focus of numerous studies [1, 2, 6].

The basic structure of class 1 integrons includes two conserved segments (CSs) that are usually separated by a variable region that includes mobile cassettes containing antibiotic resistance genes. The 5′-CS carries an integrase class 1 (*intI1*) gene encoding an integrase enzyme and a recombination site (*attI1*), whereas *qacEΔ1* and *sul1*, which confer resistance to quaternary ammonium compounds and sulfonamides, respectively, are localized at the 3′-CS [1, 6–8]. The site-specific recombination system between *attI* and *attC* has enabled a diverse array of resistance determinants to be drawn by individual class 1 integrons [1, 2, 9].

The coding regions of the gene cassettes have no promoters; however, most cassettes encode various antimicrobial resistance genes, with more than 130 distinguishable resistance genes having been found to date [6]. The majority of class 1 integrons harbors an aminoglycoside adenyltransferase gene (*aadA*) and a dihydrofolate reductase gene (*dfr*), which confer resistance to streptomycin and spectinomycin, and trimethoprim, respectively [10, 11].

Tetracycline has been used in human and veterinary medicine and as a growth promoter in animal husbandry. The major mechanisms of tetracycline resistance in *Enterobacteriaceae* are mediated through one of several mechanisms; namely, efflux pump activity, ribosomal protection, and enzymatic inactivation. The predominant genes that confer tetracycline resistance via efflux pump activity are *tet(A)*, *tet(B)*, *tet(C)*, *tet(D)*, *tet(E)*, and *tet(G)*. Indiscriminate application of tetracyclines in food-producing animals enhances multidrug resistance due to antibiotic selective pressure induced by the presence of high environmental concentrations of the antibiotics. This selective pressure ultimately leads to increased prevalence of tetracycline resistance via the *tet* genes and promotes the dissemination of mobile genetic elements in bacteria [12, 13].

*E. coli* isolates belong to four major phylogenetic groups, A, B1, B2, and D, and strains from the B2 phylogenetic group happen to be the least resistant to antimicrobials. Moreover, there is a tendency towards lower integron carriage among phylogroup B2 [14]. Nevertheless, due to certain factors, such as the level of resistance to antimicrobials, the site of the infection and geographical location, there is variation in the prevalence of different phylogenetic groups [15]. Isolates in the phylogenetic groups B1 and D tend to harbor class 1 integrons, and a previous report also showed that *intI*-positive B2 strains were the least prevalent [16]. There are various observations on the interrelationship of different phylogroups and integron carriage for environmental, human and animal isolates raising the hypothesis that the two phenomena are connected and indicating that various genetic elements are involved in strains with different phenotypes. Characterization of this association will help to better understand the infection process and will reflect the possible different survival strategies of *E. coli* phylogroups under different circumstances.

Antimicrobial resistant bacteria derived from animals seriously compromises public health by causing food-borne infections and raises a food safety issue globally. The effects of such bacteria are not only limited to food safety but also pose occupational hazards for animal handlers, meat inspectors and veterinarians. In particular, carriage of antibiotic-resistant foodborne pathogens by food-production animals is one of many contributors to treatment failure in health care settings, and it establishes the need for a detailed and thorough investigation of the carriage of such antibiotic-resistant pathogens harboring integrons in food-producing animals [12]. Furthermore, integrons are not only limited to pathogenic organisms but have also been isolated from bacteria recovered from environmental samples and healthy animals [17]. Similarly, the lack of sufficient and current information describing the association between antibiotic resistance and phylogenic groups with respect to integron carriage in commensal *E. coli* isolates of cattle from Korea necessitates further research. Therefore, in the present study, we investigated the role of integrons and their associated diverse gene cassettes in mediating antimicrobial resistance in commensal *E. coli* isolates recovered from cattle. Moreover, we examined the relationship between class 1 integron carriage with respect to phylogroups and patterns of tetracycline resistance.

## Methods

### Selection of bacterial strains for the study

In total, 247 commensal *E. coli* isolates obtained by our research group between 2014 and 2015 from fecal samples from 405 tested animals at four healthy beef cattle farms located in four different cities in South Korea (Pyeongchang, Anyang, Yangpyeong, and Cheonan) were used in the present study [18]. The beef farms consisted of different age groups of cattle, such as weaned calves, bulls and steers. Since the farms are intensive, cattle were kept in confinement in a conventional housing system. Fecal samples were freshly collected from the rectum of each cattle and a single bacterial isolate was recovered per animal. All bacterial strains were routinely cultured in tryptic soy broth (TSB) (Oxoid, Basingstoke, UK) for 18 h at 37 °C. Among the 247 isolates, 92 *E. coli* isolates demonstrating resistance or decreased susceptibility by microbroth dilution assays to any of the tetracycline antibiotics referred to below were selected for further investigation.

### Susceptibility testing

Phenotypic characterization for all isolates was performed using the disc diffusion method, and the following antibiotic discs were analyzed in this study: tetracycline (TE, 30 μg), streptomycin (S, 10 μg), chloramphenicol (C, 30 μg), ampicillin (AMP, 10 μg), amoxicillin-clavulanic acid (AMC, 30 μg), ciprofloxacin (CIP, 5 μg), nalidixic acid (NA, 30 μg) and trimethoprim-sulfamethoxazole (SXT, 25 μg) (Sigma-Aldrich, St. Louis, MO, USA). The MICs for oxytetracycline, doxycycline, tetracycline, minocycline and tigecycline were determined using cation-adjusted Mueller-Hinton broth (Oxoid, Basingstoke, UK). All susceptibility testing was performed according to the procedures and interpretive criteria specified by the Clinical Laboratory Standards Institute (CLSI), and *E. coli* ATCC 25922 was used as a quality control strain [19].

### Phylogenetic group determination

*E. coli* phylogenetic groups (A, B1, B2 and D) were investigated by amplifying two genes and a DNA fragment using multiplex PCR as previously described [20].

### Analysis of antimicrobial resistance genes

PCR amplification to investigate the tetracycline resistance-encoding genes was conducted for all isolates. The following genes, encoding the tetracycline efflux mechanism, were investigated as previously described: *tet(A)*, *tet(B)*, *tet(C)*, *tet(D)*, *tet(E)* and *tet(G)* [12, 21–24]. Furthermore, genes conferring resistance to sulfonamide antibiotics (*sul1*, *sul2* and *sul3*) and genes conferring chloramphenicol/florfenicol resistance (*cat1*, *cmlA* and *floR*) were also analyzed. PCR amplification of the resistance genes was conducted using the primers presented in Table 1 [12, 21–30].

**Table 1:** Primers used for the PCR detection of resistance genes

Primer name	Target gene	Nucleotide sequence	Annealing temperature (°C)	Amplicon size (bp)	Reference
TetA-F	*tet(A)*	GGCGGTCTTCTTCATCATGC	55	502	[21]
TetA-R		CGGCAGGCAGAGCAAGTAGA			
TetB-F	*tet(B)*	CATTAATAGGCGCATCGCTG	55	930	[21]
TetB-R		TGAAGGTCATCGATAGCAGG			
TetC-F	*tet(C)*	GCTGTAGGCATAGGCTTGGT	55	888	[21]
TetC-R		GCCGGAAGCGAGAAGAATCA			
TetD-F	*tet(D)*	GAGCGTACCGCCTGGTTC	55	780	[12]
TetD-R		TCTGATCAGCAGACAGATTGC			
TetE-F	*tet(E)*	AAACCACATCCTCCATACGC	55	278	[22]
TetE-R		AAATAGGCCACAACCGTCAG			
TetG-F	*tet(G)*	GCTCGGTGGTATCTCTGCTC	55	468	[23]
TetG-R		AGCAACAGAATCGGGAACAC			
Sul1-F	*sul1*	CGGCGTGGGCTACCTGAACG	57	433	[24]
Sul1-R		GCCGATCGCGTGAAGTTCCG			
Sul2-F	*sul2*	CGGCATCGTCAACATAACCT	57	721	[21]
Sul2-R		TGTGCGGATGAAGTCAGCTC			
Sul3-F	*sul3*	CAACGGAAGTGGGCGTTGTGGA	57	244	[25]
Sul3-R		GCTGCACCAATTCGCTGAACG			
Cat-F	*Cat*	GGT GAG CTG GTG ATA TGG	55	209	[26]
Cat-R		GGG ATT GGC TGA GAC GA			
Flor -F	*flor*	CAC GTT GAG CCT CTA TAT	55	868	[27]
Flor -R		ATG CAG AAG TAG AAC GCG			
CmlA -F	*cmlA*	TGT CAT TTA CGG CAT ACT CG	55	455	[27]
CmlA -F		ATC AGG CAT CCC ATT CCC AT			
Var1-F	*var1*	GGCATCCAAGCAGCAAG	55	Variable	[28]
Var1-R		AAGCAGACTTGACCTGA			
qacEΔ1 F	*qacEΔ1*	ATCGCAATAGTTGGCGAAGT	60	225	[29]
qacEΔ1 R		CAAGCTTTTGCCCATGAAGC			
IntI1-F	*intI1*	GGGTCAAGGATCTGGATTTCG	60	483	[30]
IntI1-R		ACATGCGTGTAAATCATCGTCG			

### Detection and characterization of class 1 integrons and their gene cassettes

Total DNA was extracted by boiling a suspension of overnight-cultured bacterial cells [grown on tryptic soy agar plates (TSA) at 37 °C for 10 min] in 200 μl of sterile RNase/DNase-free distilled water. All *E. coli* isolates were PCR screened for the presence of *IntI1* gene-encoding class 1 integrons. Further testing was performed on the integron positive isolates for the presence of gene cassettes in the variable region and the *sul1* and *qacE∆1* genes in the 3′-CS. All primers and PCR conditions are presented in Table 1. Gel purification of all PCR products was conducted using PCR quick-spin PCR product purification kits (iNtRON Biotechnology, USA), after which the samples were sequenced (Macrogen Co., Seoul, Korea). Following sequencing, the gene cassettes within the variable regions of the class 1 integrons were determined by using BLAST (Basic Local Alignment Search Tool) searches of the NCBI database (National Center for Biotechnology Information).

### Clonal relationships among integron positive strains

Determination of the genetic relationship between the integron positive isolates was accomplished by pulsed-field gel electrophoresis (PFGE) analysis according to the protocols and criteria previously established by the Centers for Disease Control and Prevention (CDC) using *XbaI* as the restriction enzyme. Briefly, following 18 to 20 h growth on TSA at 37 °C, genomic DNA was digested with 50 U *XbaI* (TaKaRa, Japan) for 2 h at 37 °C, then the DNA fragments were subsequently separated on a 1.0% SeaKem Gold agarose gel (Lonza, USA) in 0.5× Tris-borate-EDTA (TBE) buffer using a CHEFMapper gel apparatus (Bio-Rad Laboratories, California, USA). The conditions for electrophoresis were as follows: pulse time, 2–30s at 14 °C; run time, 18 h; voltage, 6 V/cm. Analysis of the image was performed by using the Bionumerics software (Applied Maths, Belgium).

### Statistical analysis

All experiment data are stored in Excel 2010, and the susceptibility testing was analyzed using IBM SPSS/Statistics, version 24. The association between the *tet* genes and the presence of class 1 integron gene was analyzed by Fisher’s exact test or Pearson’s χ^2^ test, contingent on cell frequencies. The median MICs for the respective tetracycline antibiotics between the isolates with and without *Intl1* was analyzed using the Mann-Whitney test. A *P* < 0.05 was considered to indicate statistical significance.

## Results

### Antimicrobial resistance phenotypes

**Fig. 1:**
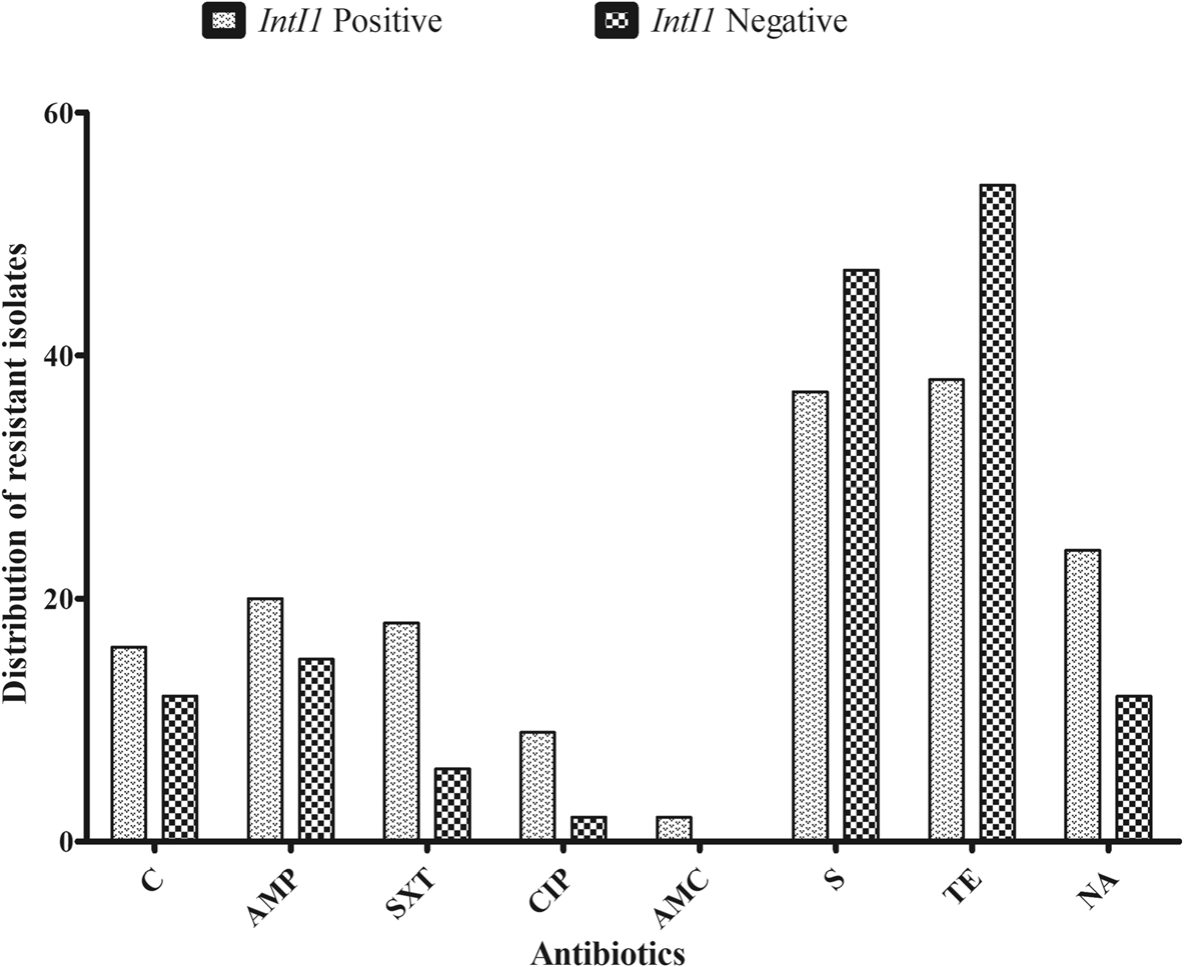
Different classes of antibiotics with respect to their presence or absence in integrons

The resistance percentages to the tested antibiotics were as follows: streptomycin, 84 (91.3%); nalidixic acid, 36 (39.1%); ampicillin, 35 (38%); chloramphenicol, 28 (30.4%); trimethoprim-sulfamethoxazole, 24 (26.1%); ciprofloxacin, 11 (12%) and amoxicillin-clavulanic, 2 (2.2%) (Fig. 1). The MIC range for the 92 tetracycline resistant isolates was > 256 μg/ml to 16 μg/ml, and their MIC_50_ and MIC_90_ values were 128 and 256 μg/ml, respectively. Oxytetracycline resistance was identified in all isolates (MIC range > 256 μg/ml–32 μg/ml), of which 49 isolates were highly resistant (MIC ≥256 μg/ml). Moreover, 80 strains (87%) were resistant to doxycycline, 41 (44.6%) to minocycline and none to tigecycline. Significantly higher median oxytetracycline MICs were observed for isolates with class 1 integrons than for isolates without class 1 integrons (*P* < 0.006; Table 2); however, there were no significant statistical differences in the median MIC values for tetracycline, doxycycline, minocycline and tigecycline between class 1 integron-positive and integron-negative strains.

**Table 2:** Susceptibility to various tetracycline antibiotics stratified by the presence or absence of class 1 integrons

Antimicrobial agents	*Intl1* present					*Intl1* Absent					*P* value
	MIC (μg/ml)				Number (%) of resistant isolates	MIC (μg/ml)				Number (%) of resistant isolates	
	Range		MIC_50_	MIC_90_		Range		MIC_50_	MIC_90_		
Tetracycline	64	> 256	128	256	38 (41.3)	16	> 256	128	256	54 (58.7)	0.244
Doxycycline	4	128	16	64	36 (39.1)	4	64	32	64	44 (47.8)	0.975
Oxytetracycline	64	> 256	> 256	> 256	38 (41.3)	32	> 256	256	> 256	54 (58.7)	*P<0.006*
Minocycline	4	64	8	32	14 (15.2)	2	64	16	32	28 (30.4)	0.267
Tigecycline	0.25	2	1	1	–	0.25	8	0.5	1	1 (1.1)	0.054

Numbers indicated in italics indicate significance difference (*P*< 0.05)

### *E. coli* phylogenetic groups

**Fig. 2:**
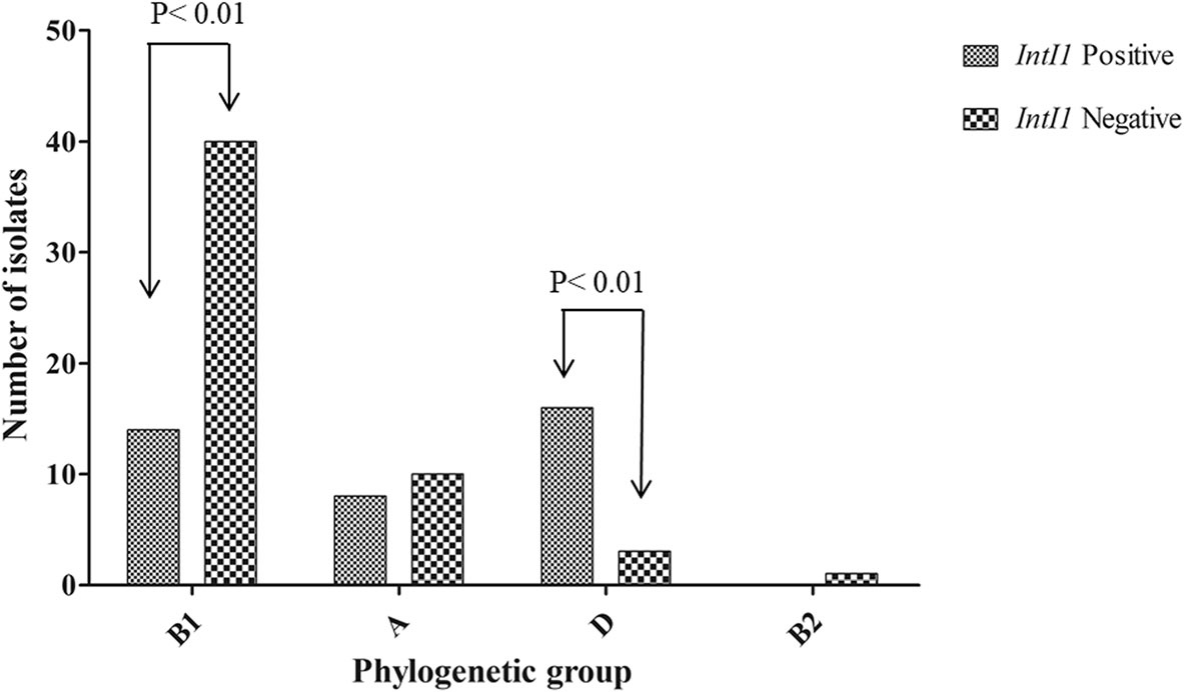
Association between phylogenetic group and isolates carrying integrons

Of the 92 isolates, phylogenetic groups B1 and D were the most common (54 isolates; 58.7% and 19 isolates; 20.7%, respectively), followed by group A, which was assigned to 18 isolates (19.6%). Group B2 was rare, occurring in only 1 isolate. We also compared integron-positive and integron-negative isolates across the phylogenetic groups, and phylogenetic group D (16 isolates) was the most prevalent among the *intI1*-positive isolates, whereas B1 (40 isolates) was most prevalent among the *intI1*-negative isolates. Our results showed an association between the presences of class 1 integrons and affiliation with phylogenetic groups D and B1 (*P* < 0.01). The frequencies of integron-negative and integron-positive strains for in A and B2 phylogenetic groups were similar, with no statistically significant differences (Fig. 2).

### Characterization of antimicrobial resistance genes

All isolates carried at least one of the *tet* genes examined. Overall, 88 (95.7%) of the tetracycline-resistant isolates carried *tet(A)* or *tet(B)*, with 47 (51.1%) and 41 (44.6%) isolates harboring only *tet(A)* or *tet(B)*, respectively. The *tet(C)* and *tet(G)* genes were found in only five (5.4%) and six (6.5%) isolates, respectively, and the *tet(C)* gene was found in isolates that were not harboring integrons. Moreover, eight isolates harbored two *tet* genes, while none of the isolates carried the *tet(D)* or *tet(E)* genes. The distribution of tetracycline resistance genes among the integron-positive and -negative isolates is shown in Table 3. *E. coli* isolates carrying class 1 integrons were more likely to harbor the *tet(A)* gene (*P* < 0.01). In addition, the following determinants for chloramphenicol/florfenicol resistance were identified: *cat1* (47.4%), *floR* (50%), and *cmlA* (18.4%). Among the 92 *E. coli* isolates investigated, 28 isolates harboring integrons and eight isolates without integrons (*n* = 36; 39.1%) had the *sul1* gene. Moreover, the *sul2* and *sul3* genes were identified in 16 (17.4%) and seven (7.6%) isolates, respectively.

**Table 3:** Association between integron-positive and integron-negative *E. coli* isolates and the frequencies of *tet* genes

*tet* genes	Class 1 integron presence		*P* value
	*intI1* positive	*intI1* negative	
*tet(A)*	26	21	*P < 0.01*
*tet(B)*	12	30	*0.023*
*tet(C)*	–	5	0.054
*tet(G)*	1	5	0.205
*tet(A) + tet (B)*	–	1	0.399
*tet(A) + tet (C)*	–	1	0.399
*tet(B) + tet (G)*	1	5	0.205

Numbers indicated in italics indicate significance difference (*P*< 0.05)

### Detection of the *intI1* gene and characterization of gene cassettes in the *E. coli* strains

Integrase gene-encoding class 1 integrons were detected by PCR in 38 (41.3%) isolates. Resistance to quaternary ammonium compounds and sulfonamides conferred by the *qacE∆1* and *sul1* genes in the 3′-CS, respectively, was identified in 36 integron-positive isolates. Among these, 27 contained the entire 3′-CS (*qacE∆1*-*sul1*) structure, whereas nonclassical integrons lacking the 3′-CS were found in only two of the 38 *intI1*-positive *E. coli* isolates. Of the 38 isolates, one had only *sul1* in the 3′-CS and eight possessed only *qacE∆1* in the 3′-CS. The class 1 integron variable regions were amplified in 32 (84.2%) of the 38 *intI1*-positive isolates, and their genetic contents were ascertained via PCR amplification of the integron variable regions and subsequent full sequence analysis. Different lengths of PCR products ranging from ~ 1–2.5 kb were observed for strains having variable regions. Of these, the predominant cassette amplicons carried by the isolates were 1 kb in 18 strains, 1.5 kb in 10 strains and 2.5 kb in four strains (Table 4).

**Table 4:** Characterization of *E. coli* isolates harboring class 1 integrons and description of their associated gene cassettes

Isolates No.	3’CS	Cassette amplicons (bp)	Other resistance gene pattern	Integron gene cassette arrays	PFGE pattern	Resistance pattern
EC151	*qacE∆1-sul1*	1500	*tetA, sul1, sul2, cat1*	*aadA1-dfrA1*	B	TE-S-C-SXT
EC139	*qacE∆1-sul1*	1500	*tetB, tetG, sul1, sul2, cat1, floR*	*aadA1-dfrA1*	A	TE-S-C-AMP-SXT-CIP-NA
EC143	–	1500	*tetA, sul3, cmlA, cat1*	*aadA1-dfrA12*	U	TE-S-AMP-SXT-CIP-NA
EC147	*qacE∆1-sul1*	1000	*tetA, sul1*	*aadA2*	H	TE-S-NA
EC152	*qacE∆1-sul1*	1000	*tetA, sul1*	*aadA1*	H	TE-S-AMP-SXT-NA
EC153	*qacE∆1-sul1*	2500	*tetA, sul1*	*aadA1-aadA2-dfrA12*	H	TE-S-NA
EC155	qacE∆1-sul1	–	*tetB, sul1, sul2, cat1*	*–*	S	TE-S-C-SXT-CIP-NA
EC156	*qacE∆1-sul1*	1000	*tetA, sul1, floR*	*aadA1*	H	TE-S-AMP-NA
EC157	*qacE∆1-sul1*	1500	*tetB, sul1, sul2, cat1*	*aadA5-dfrA17*	G	TE-S-AMP-SXT-CIP-NA
EC159	*qacE∆1-sul1*	1500	*tetB, sul1, sul2, cat1*	*dfrA12*	R	TE-S-C-AMP-SXT-CIP-NA
EC160	*qacE∆1-sul1*	1500	*tetB, sul1, sul2, cat1*	*dfrA12*	R	TE-S-C-AMP-SXT-CIP-NA
EC161	–	–	*tetB, sul3, cmlA*	*–*	Q	TE-S-C
EC162	*qacE∆1-sul1*	1000	*tetA, sul1, floR*	*aadA1*	N	TE-S-NA
EC163	*qacE∆1-sul1*	1000	*tetA, sul1, floR*	*aadA1-dfrA1*	L	TE-S-NA
EC164	*qacE∆1-sul1*	1000	*tetA, sul1, floR*	*aadA1*	M	TE-S-SXT-NA
EC165	*qacE∆1-sul1*	1000	*tetB, sul1, sul2, cat1, floR*	*aadA1*	T	TE-S-C-AMP-SXT-CIP-NA
EC166	*qacE∆1-sul1*	–	*tetB, sul1, sul2, cat1*	*–*	G	TE-S-C-AMPCIP-NA
EC167	*qacE∆1-sul1*	2500	*tetA, sul1, floR*	*aadA1-aadA2-dfrA12*	K	TE-S-NA
EC172	*qacE∆1-sul1*	1000	*tetA, sul1, floR*	*aadA1*	J	TE-S-NA
EC173	*qacE∆1-sul1*	1000	*tetA, sul1, floR*	*aadA1*	J	TE-S-NA
EC174	*qacE∆1-sul1*	1000	*tetA, sul1, floR*	*aadA1-dfrA12*	I	TE-S-NA
EC175	*qacE∆1-sul1*	1000	*tetA, sul1, floR*	*aadA1-dfrA12*	I	TE-S-NA
EC176	*qacE∆1*	1000	*tetB, sul2, sul3, cmlA, floR*	*aadA1*	W	TE-S-C
EC177	*qacE∆1-sul1*	1000	*tetA, sul1, floR*	*aadA1*	Y	TE-S-AMP
EC178	*qacE∆1-sul1*	2500	*tetA, sul1, floR*	*aadA1-aadA5-dfrA5*	Y	TE-S-AMP-NA
EC179	*qacE∆1*	2500	*tetB, sul2, sul3, cml1, floR*	*aad1-aadA2-dfrA12*	O	TE-S-C
EC180	*qacE∆1*	1000	*tetB, sul2, sul3, cml1, floR*	*aadA2*	P	TE-S-C
EC181	*qacE∆1-sul1*	1500	*tetB, sul1, sul2, cat1*	*aadA1-dfrA1*	D	TE-S-C-AMP-SXT-CIP-AMC-NA
EC185	*qacE∆1-sul1*	1500	*tetA, sul1, sul2*	*aadA1-dfrA1*	Z	TE-S-AMP-SXT-AMC-NA
EC191	*qacE∆1*	1000	*tetA, sul3, cml1, cat1, floR*	*aadA1-dfrA12*	A2	TE-S-C-SXT
EC194	*qacE∆1-sul1*	1500	*tetA, sul1, cat1*	*aadA1-dfrA1*	F	TE-S-AMP-SXT
EC198	*qacE∆1-sul1*	1500	*tetA, sul1, cat1*	*aadA1-dfrA1*	F	TE-S-AMP-SXT
EC209	*qacE∆1*	1000	*tetA, sul3, cml1, cat1, floR*	*aadA1-dfrA12*	X	TE-S-C-SXT
EC230	*qacE∆1*	–	*tetA, sul2, cat1*	*–*	E	TE-S-C-AMP-SXT-NA
EC231	*qacE∆1*	–	*tetA, sul2, cat1*	*–*	E	TE-S-AMP
EC254	*qacE∆1-sul1*	1000	*tetA, sul1, cat1*	*aadA1-dfrA12*	V	TE-S-*AMP*
EC258	*sul1*	–	*tetA, sul1*	*–*	A1	TE-AMP-SXT
EC262	*qacE∆1*	1000	*tetA, sul2, cat1, floR*	*aadA1*	C	TE-S-C-AMP

Overall, seven gene cassettes and eight distinct profiles of gene cassette arrays, namely, *aadA1* (10 isolates), *aadA2* (two isolates), *dfrA12* (two isolates), *aadA1-dfrA1* (seven isolates), *aadA1-dfrA12* (six isolates), *aadA5-dfrA17* (one isolate), *aadA1-aadA2-dfrA12* (three isolates) and *aadA1-aadA5-dfrA5* (one isolate), were described. The 2.5 kb amplicon consists of *aadA1*-*aadA2*-*dfrA12* and *aadA1*-*aadA5*-*dfrA5* (Table 4).

### PFGE analysis of isolates containing class 1 integrons

**Fig. 3:**
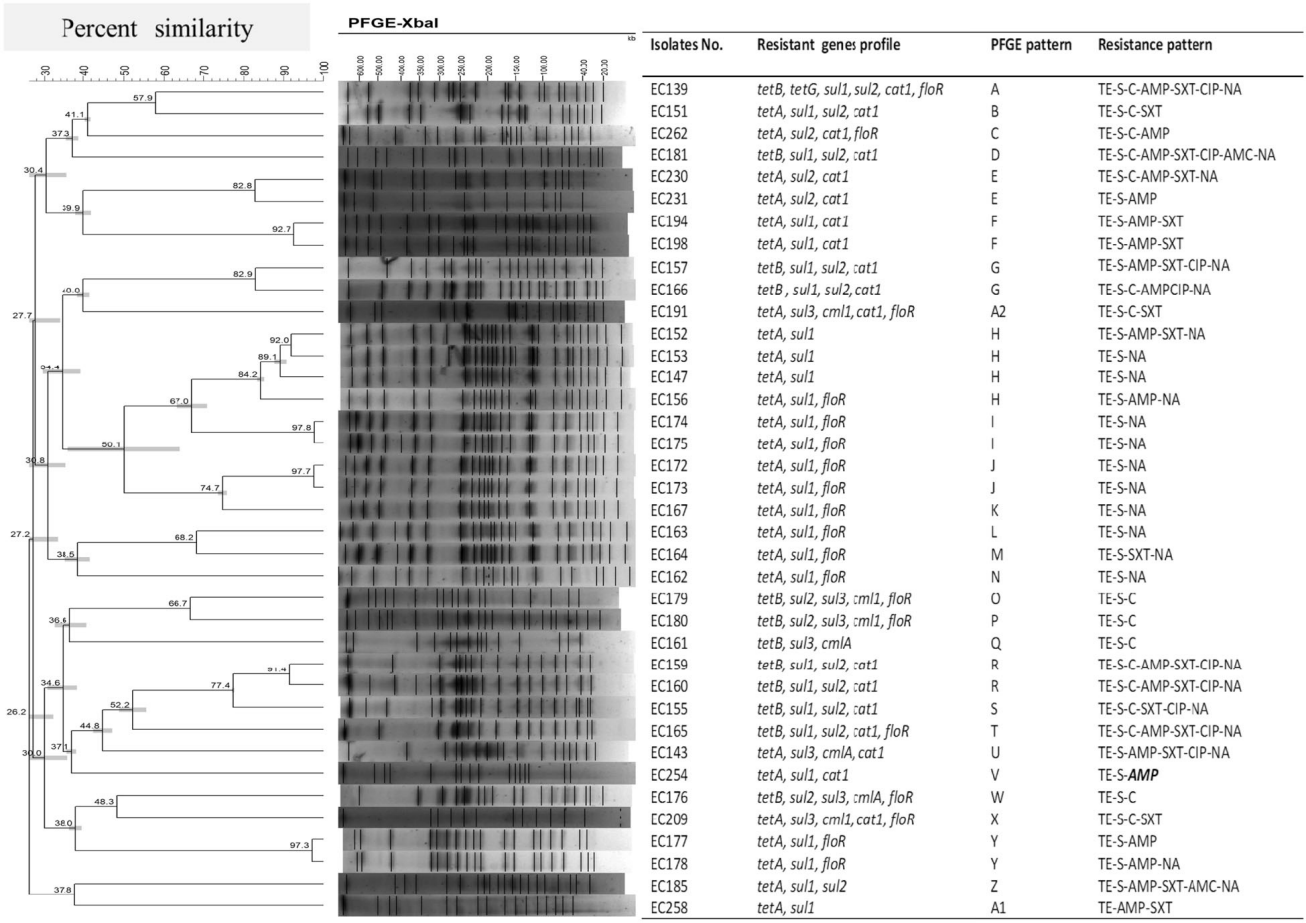
Genetic relatedness of *E. coli* isolates with class 1 integrons indicated by *XbaI*-digested chromosomal DNA

The genetic relatedness among the multidrug resistant *E. coli* isolates carrying integrons was established based on their *XbaI*-digested chromosomal DNA fragments, and the most commonly detected genotypes are depicted in Table 4 and Fig. 3. Several profiles were observed, with 12–25 discernible DNA fragments from 38 isolates when analyzed by the Dice coefficient method. When an 80% cut-off band pattern similarity was used, 28 different PFGE clusters were observed, whereas 26 clusters were detected when a 70% cut-off band pattern similarity was applied. Strong relationships (> 90% similarity) were encountered in six clusters constituting 12 isolates sharing the same antibiotics resistance spectrum and resistance gene pattern. For instance, isolates EC174 and EC175 had more than 97% band pattern similarity, as well as the same antibiotic resistance pattern (TE-S-NA), integron gene cassette arrays (*aadA1*-*dfrA12*), and resistance genes [*tet(A)*, *sul1* and *floR*].

## Discussion

Our study investigated the association of class 1 integron carriage, phylogenetic group affiliation and different tetracycline antibiotics resistance patterns in commensal *E. coli* strains isolated from cattle farms in Korea. All 92 *E. coli* isolates were significantly resistant to tetracycline and oxytetracycline. These findings indicate a widespread application of these antibiotics either for therapeutic purposes or as a supplement for promoting growth, and this continuous exposure to tetracyclines has led to a higher percentage of tetracycline-resistant *E. coli* isolates. The findings of our work were comparable with other observations where a high prevalence of resistance to antimicrobials commonly used with livestock, such as tetracycline and streptomycin were observed in commensal *E. coli* isolated from food-producing animals in South Korea. For instance, Lim et al. [31] observed tetracycline (30.5%) as the most frequently observed resistance in *E. coli* isolates of cattle origin, and Kang et al. [32] showed that *E. coli* isolates recovered from swine with diarrhea were highly resistant to streptomycin (99.0%) and tetracycline (97.1%); furthermore, the work of Shin et al. [33] also demonstrated that the most prevalent resistance phenotype observed was streptomycin (63.1%), followed by tetracycline (54.5%). Tetracycline antibiotics have long been the single most commonly used class of antimicrobial in livestock, accounting for around 50% of the total amount of antimicrobial consumption both in the USA [34] and Korea [35], and it is, therefore, not surprising to observe tetracycline resistance as the most frequently antimicrobial resistance class in *E. coli* isolates. As indicated by the animal and plant quarantine agency of Korea (APQA) [36], although gradually decreasing since 2003, tetracyclines still comprise the predominant antibiotics sold for veterinary use.

The investigated isolates were recovered from clinically healthy animals; accordingly, B1 (58.7%), which is commonly associated with nonpathogenic commensal strains, was the common phylogroup classified. Accordingly, only a single isolate was classified into phylogenetic group B2, which is normally linked with pathogenicity [37]. Moreover, no isolates categorized as B2 carried the *intI1* gene, which is similar to the results of a previous study that demonstrated that the B2 phylogroup has a lower tendency to harbor integrons than other phylogroups [16]. In the present study, significant differences in the numbers of isolates with and without integrons were observed (*P* < 0.01) between phylogroups D and B1. These agree with those of a previous study that demonstrated that strains associated with phylogroups A and B1 tend to carry integrons more often than those associated with B2 and D [10, 38]. In contrast, affiliation with a specific phylogenetic group was not linked to the presence of integrons in *E. coli* strains recovered from river water [39]. This variability is likely because of ecological differences among the sites from which the *E. coli* isolates were recovered that may influence their ability to harbor integron genes.

In the present study, the *tet(A)* gene was the predominant resistance determinant, followed by the *tet(B)* gene. There is general agreement regarding the widespread importance of the link between the *tet(A)* and *tet(B)* genes and resistance to tetracycline antibiotics in *Enterobacteriaceae* as reported by multiple investigators [12, 40, 41]. In this study, isolates having more than one *tet* gene were also observed in 8.7% of the strains, which is a common phenomenon in *E. coli* isolates of cattle origin. Previous studies have shown similar results, in which 3.5% [42], 5.4% [43] and 22.2% [44] of isolates had two *tet* genes, with only a slight difference in the total number of isolates used between the studies. The acquisition of more than one *tet* gene by a given strain is attributed to powerful selection pressures due to the high level of tetracycline in the environment rather than to a special selective advantage conferred by the *tet* genes [12].

There are varying accounts of which *tet* gene is most frequently reported in different countries. For example, Karami et al. [13] reported that *tet(B)* was the most frequently observed (51%) among commensal *E. coli* strains from Sweden, while Shin et al. [41] and Dessie et al. [45] reported that *tet(A)* accounted for 46.5% and 63.2% of all *tet* genes detected in Korea, respectively. A significantly higher frequency of the *tet(A)* gene (*P* < 0.01) was also observed in isolates with integrons, demonstrating an association between *tet(A)* carriage and presence of class 1 integrons. This observation has previously been reported by others, who found that *intI1* and *tet(A)* coexisted on the same large transferable plasmid or other genetic elements in *E. coli*, validating an established association between tetracycline resistance genes and class 1 integrons [46, 47]. *sul1* was identified in 39.1% of isolates; since it is commonly linked to integrons and transposons as a component of the 3′-CS, previous studies have similarly reported it among bacteria of the family *Enterobacteriaceae* [48]*.*

In the present study, 41.3% of *E. coli* isolates harbored *intI1* gene-encoding class 1 integrons. A comparable result regarding the prevalence of class 1 integrons was previously reported in Korea and other countries; for instance, 40% of the *E. coli* isolates carried class 1 integrons in Lithuania [49], 49.8% in Italy [50], and 27% in the United States [51], as well as 44% of the commensal *E. coli* isolates from poultry in Korea [52]. Non-classical integrons lacking the normal 3´-CS were detected in only two class 1 integron-positive isolates. Similar observations were made for *intI1*-positive *E. coli* isolates that originated from food, animals, and healthy humans [53]. Moreover, 32 (84.2%) of the 38 *intI1*-positive isolates had variable regions containing gene cassettes. Overall, our analysis showed that the *aad* and *dfr* families comprise the majority of class 1 integron gene cassettes, similar to the results reported for *E. coli* originating from beef cattle [38]. In the present study, *aadA1*-*dfrA1* was the most commonly detected combination, which is in agreement with previous reports on isolates recovered from clinical and healthy animals, humans and food samples [27, 49, 52, 54]. Furthermore, 27 (71.1%) of the cassette arrays contained the *aadA1* gene, either alone or in combination with other gene cassette arrays that encode aminoglycoside adenyltransferases, which confer resistance to streptomycin/spectinomycin [39]. When we made a comparison between these environmental isolates and clinical isolates from the same region, commensal *E. coli* isolates from animals mostly carried a single gene cassette, whereas clinical *E. coli* isolates from humans had multigene cassettes [52]. In addition, we found between one and three gene cassettes in a single isolate, which is a distinguishing feature of class 1 integrons in which no more than 6 gene cassettes are carried in the variable region [4].

Based on the results of the *XbaI*-PFGE, the *E. coli* isolates carrying class 1 integrons could be categorized into 28 and 26 different PFGE cluster groups when 80% and 70% cut-off band pattern similarities were applied, respectively. In this study, *E. coli* isolates carrying integrons showed a high degree of polymorphism. This diverse clonal relationship resulted from the horizontal transfer of resistance genes between different strains, rather than a dissemination of a single clonal strain, as previously described [55].

## Conclusions

Due to their carriage of resistant genes and class 1 integrons, commensal *E. coli* isolates have a significant implication in public health through their ability to disseminate antibiotic resistant genes via contamination of the food chain. A positive association was observed between isolates harboring the *intI1* and *tet(A)* genes, confirming that isolates containing the *tet(A)* gene are more likely to carry class 1 integrons. Likewise, affiliation with phylogroup D was positively associated with the presence of class 1 integrons. Further detailed investigation of the class 1 integron genetic content should be conducted to provide a more complete understanding of the molecular mechanisms responsible for multidrug resistance in *E. coli* strains. Moreover, the interrelation of integron related resistance genes with other factors should be studied by integrating environmental and veterinary factors and factors associated with the food chain. Accordingly, the resulting advances could have a profound effect on clinical practice, infection control measures and treatment options, both in veterinary and human medicine.

## Abbreviations

APQA: Animal and plant quarantine agency of Korea
CS: Conserved segment
KFDA: Korean Food and Drug Administration
MIC: Minimum inhibitory concentration
PCR: Polymerase chain reaction
PFGE: Pulsed-field gel electrophoresis
USFDA: United States Food and Drug Administration
